# Bridging HIV-1 Cellular Latency and Clinical Long-Term Non-Progressor: An Interactomic View

**DOI:** 10.1371/journal.pone.0055791

**Published:** 2013-02-25

**Authors:** Jin Yang, Zongxing Yang, Hangjun Lv, Yi Lou, Juan Wang, Nanping Wu

**Affiliations:** 1 State Key Laboratory for Diagnosis and Treatment of Infectious Diseases, Institute of Infectious Diseases, The First Affiliated Hospital of Zhejiang University, School of Medicine, Zhejiang University, Hangzhou, China; 2 Department of Medicine, Blood Center of Zhejiang Province, Hangzhou, China; 3 Department of Medicine, School of Medicine, Hangzhou Normal University, Hangzhou, China; George Mason University, United States of America

## Abstract

Development of an effective HIV management is enticed by the fact that long-term non-progressors (LTNP) restrict viral replication spontaneously, but is hindered by HIV-1 latency. Given that the most overlapping characteristics found between HIV-1 LTNP and latency, detailed analysis of the difference would disclose the essentials of latency. In this study, microarray data from our previous study was combined with HIV-1 latency and LTNP data obtained from NCBI GEO database. Principal variance component analysis and hierarchical clustering verified the removal of batch effect across platform. The analysis revealed a total of 456 differential expressed genes with >2-fold change and B-statistic >0. Bayesian inference was used to reconstitute the transcriptional network of HIV-1 latency or LTNP, respectively. Gene regulation was reprogrammed under different disease condition. By network interference, KPNA2 and ATP5G3 were identified as the hubs in latency network which mediate nuclear export and RNA processing. These data offer comparative insights into HIV-1 latency, which will facilitate the understanding of the genetic basis of HIV-1 latency *in vivo* and serve as a clue for future treatment dealing with key targets in HIV-1 latency.

## Introduction

An definition of viral latency reflects a state of reversibly nonproductive infection of individual cells [1]. For human immunodeficiency virus-1 (HIV-1), the term latency was initially used in the clinical sense to describe the long asymptomatic period between initial infection and the development of acquired immunodeficiency disease (AIDS). Studies revealed that after initial infection, HIV-1 establishes a persistent latent reservoir in resting CD4 T cells and other cell types in all infected subjects [2]. With time, additional epigenetic mechanisms may enforce latency [3]. Residing in the latent state, the virus persists simply as genetic information, and is thus unaffected by antiretroviral therapy (ART) or immune responses [1]. Latency cells become a major barrier to HIV-1 eradication [4].

Hope for the development of an effective HIV management is enticed by the ability of a small proportion of HIV-infected individuals to spontaneously control HIV replication [5]. These patients, called long-term non-progressor (LTNP) or more specifically elite controller, maintain undetectable levels of viral replication in the absence of ART [5]. Criteria for LTNP could be reviewed elsewhere [6], [7]. These patients have moved into the center of current efforts to identify effective host defense mechanisms against HIV.

Indeed, residual viremia, which reflects the persistent viremia at levels below the sensitivity threshold of the standard clinical assay (50 copies/ml), could be observed in LTNP patients [8]. Direct analysis of residual viremia has provided little evidence for the notion that these viruses are derived from ongoing productive rounds of viral replication, and a line of evidences also showed that intensification studies did not reduce residual viremia or even the size of the latent reservoir [9], [10].

Though the terms latency and reservoir have been used rather loosely [1], a practical definition for HIV latency is used at the cellular level, whereas LTNP is described at the individual level in a clinical sense. From the view of infection dynamic, a connection between latent infection and LTNP seemed quite likely.

However, studying latently infected cells from HIV-infected subjects is challenging, since these cells are very rare in the blood, and there are no biomarkers and methods to enrich them. To date, the best-characterized models of HIV latency involve immortalized T-cell lines [2], [11], which presented the relationship between T-cell stimulation and proviral reactivation [12], but they could not recapitulate the non-dividing state of resting CD^4+^ T cells *in vivo*. A series of latency model using primary T cells have been developed [13], [14].Unfortunately, most primary cell models used one or more rounds of cellular stimulation in the presence of specific cytokines. The process often needed several weeks or months of continuous culture, and too few cells have transitioned to a quiescent state that can be used for further study [11]. Recent reports also suggest latent reservoir *in vivo* might be more complex than thought [1], thus making the phenotype of HIV-1 infection in clinical setting even more complicated.

Given that the most overlapping characteristics, i.e. undetectable or residual viremia, and HIV-1 replication restriction found between HIV-1 LTNPs with HIV-1 latency, detailed analysis of the difference between two groups would gain novel insights into the molecular mechanisms governing HIV-1 latency. In this study, we combine the relevant microarray data sets to increase statistical power to detect biological difference between latency and LTNP. Moreover, the disease specific gene regulatory network is inferred and analyzed to define the corresponding biological process.

## Results

### Microarray data merging validation

**Figure 1:**
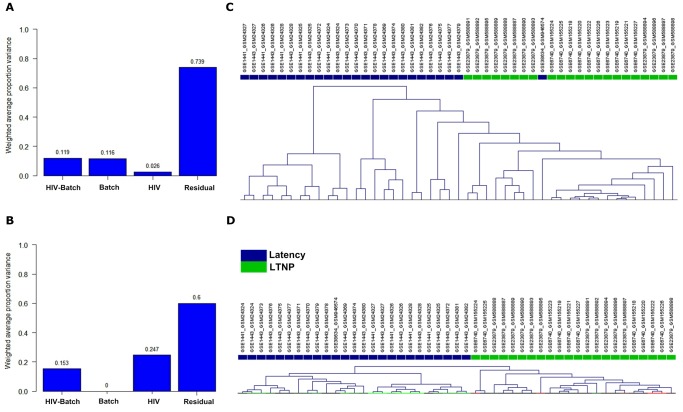
Microarray data merging. All the effects, including batch effects, profile effects, interaction between batch and profile effects, and residual effects, were estimated for their contribution to the overall variation by PVCA. (**A**) Data before batch adjustment. (**B**) Data processed by ComBat as batch adjustment tool/model. (**C**) Hierarchical clustering of the data before batch adjustment. (**D**) Hierarchical clustering of the data after batch adjustment.

With the aim to identify HIV-1 latency related markers at the genome level, all the available microarray data sets relevant to HIV-1 latency or LTNP in NCBI GEO database were merged into a composite dataset, which consisted of 25 latency and 22 LTNP samples. To assess the quantity of batch effect derived from different assays, a hybrid approach known as principal variance component analysis (PVCA) was performed, which reveals intermixing of samples from different sources before and after adjustment [15]. The PVCA revealed that batch effects explained 11.6% of the overall variation in the original data (**Fig. 1A**). After applying ComBat to remove batch effect across the different platform [16], the variation was completely eliminated (**Fig. 1B**).

The results of hierarchical clustering analysis before and after batch adjustment were also presented (**Fig. 1C and D**). Sample clustering showed a separation of the two groups of samples where adjustment for batch effects was not performed. After batch adjustment, the clusters were no longer confused with the batch effects.

### Identification of HIV latency related differential expressed (DE) genes

Differences in gene expression were measured with LIMMA analysis [17], [18]. The analysis revealed a total of 456 DEs with >2-fold change and B-statistic >0 between two groups (Supplementary Table S1). 212 genes were up-regulated and 244 down-regulated in latency state, indicating a relatively higher cellular activity in LTNP state.

**Figure 2:**
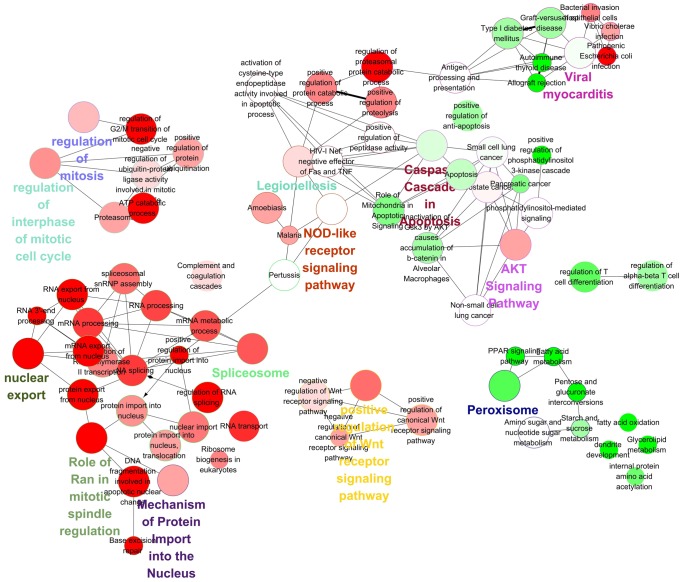
Function analysis of DEs. Terms with latency or LTNP up-regulated genes is shown in red/green, respectively. The size of the nodes reflects the statistical significance of the terms. The degree of connectivity between terms is calculated using kappa statistics. The calculated kappa score is also used for defining functional groups. The group leading term is the most significant term of the group. The color gradient shows the gene proportion of each group associated with the term. Equal proportions of the two groups are represented in white. Double-sided hypergeometric test yielded the enrichment for GO terms. Benjamini-Hochberg correction for multiple testing controlled the P-values. GO term fusion was applied for redundancy reduction.

To facilitate the understanding of the biological implications of the DE genes, function enrichments were performed by using ClueGO, which incorporates gene-ontology and KEGG/Reactome/BioCarta pathway annotation (**Fig. 2**). The distribution of any combination of terms between up- and down-regulated genes can be simultaneously tested in ClueGO [19].

Some differences emerged in gene expression between latency and LTNP group that may be relevant to the pathogenesis of the infection. Antigen processing and presentation, NOD-like receptor signaling pathway, and phosphatidylinositol-mediated signaling were present in both groups. Most of the up-regulated genes in latently infected cells were related to mRNA metabolic process, especially to the nuclear export (BAMBI, KPNA2, KPNB1, and RAN, etc), RNA splicing (DDX39A, SF3A3, SF3B4, SNRPB, and TGS1, etc) and spliceosome (ACIN1, AKT1, HNRNPH1, and HNRNPU, etc). Many up-regulated genes were also involved in cell differentiation and cell cycle regulation, such as regulation of interphase of mitotic cell cycle(ATP5H,BCL6,BRCA1, CCND1,CDC20,CDC6,and VCP, etc), and role of Ran in mitotic spindle regulation(APEX1,FEN1,HMGB1,KPNA2,KPNB1,RAN, and SFRP1). Furthermore, the remaining up-regulated genes were related to AKT signaling pathway (AKT1, BUB1B, CCND1, CCNE1, ERBB3, FOXO1, FOXO4, PIK3CA, and TGFA, etc), and Wnt receptor signaling pathway (BAMBI, CAPRIN2, CAV1, and TAX1BP3, etc). On the other hand, up-regulated genes in LTNPs were mostly associated with functions like regulation of T cell differentiation (ANXA3, CALR, DOCK1, IL23A, TGFBR2, and TNFSF4), peroxisome (ACOX3, ALDH3A2, CYP1B1, and UGDH, etc), as well as caspase cascade in apoptosis (ARHGDIB, BIRC2, BIRC3, HSP90AB1, IL1B, LAMA3, and TGFBR2, etc).

### Gene regulatory network inference

**Figure 3:**
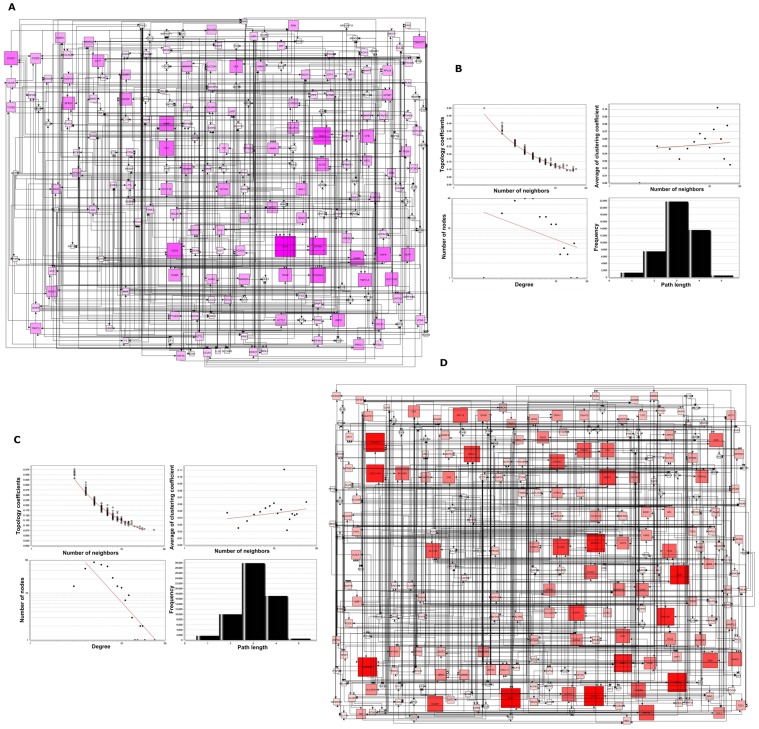
Gene regulatory network. (**A**) The HIV latency network was inferred for 212 HIV latency over-expressed genes from all latency samples. Standard Banjo parameters were adopted with a q6 discretization policy. The consensus graph depicted here was obtained from the concensus of the best 100 nets. The node size was proportional to the betweeness centrality and visually reinforced. (**B**) Topological coefficient measures the extent to which a gene in the network shares interaction partners with other genes. Average clustering coefficient measures the degree to which genes in the network tend to cluster together. The node distribution degree gives information of the protein interactions with the other genes, which shows a scale free property in this network. The shortest path length distribution indicates that the network possesses small-world property. (**C**) Topological parameters of LTNP network. (**D**) LTNP network was generated by 244 HIV LTNP over-expressed genes from all HIV LTNP samples.

**Figure 4:**
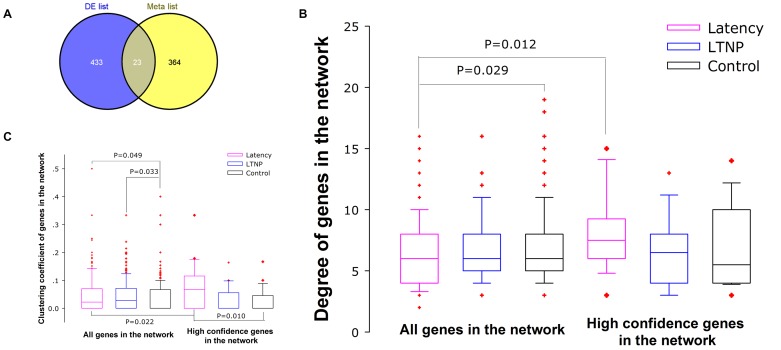
Distinct topological features of disease genes. (**A**) Overlapping analysis revealed 23 coincident HIV disease genes that have been found in global siRNA screen assays. These genes are high confident disease genes. (**B**) The difference in degrees between disease genes and all genes under latency, LTNP or normal control condition. (**C**) Clustering coefficient of disease genes in three groups. P-values are calculated using the Wilcoxon rank-sum test.

Complexity of genetic regulatory mechanisms in viral-host interaction is thought to be achieved through controlled and coordinated network. We applied Banjo to infer the Bayesian structure, since this method was better at recovering the gene networks as compared to the other approaches in recent studies [20]. 212 over-expressed latency DEs were used to generate the latency network from all latency samples (**Fig. 3**), and the network for LTNP and normal control state (named latency genes-LTNP state and latency genes-control state, respectively, see Supplementary Figs. S1 and S2). On the contrary, 244 over-expressed LTNP DEs were used to generate the LTNP network (**Fig. 4**) and the network for latency and normal control state, respectively (Supplementary Figs. S3 and S4). The latency network has 212 nodes and 677edges (interactions), and the LTNP network has 244 nodes and 841 edges.

Even though the network was solely based on transcription profiles, the inferred gene regulatory networks recapitulated many previously known molecular associations. For latency over-expressed genes, 50/677, 33/693, and 37/738 interactions in the latency network, latency genes-LTNP state network and latency genes-control state network respectively, were found in ConsensusPathDB, which integrates several protein-protein interaction database such as BioGrid, MINT, DIP, et al [21], [22]. For example, latency network predicted targeting interactions that are in agreement with previously reported biochemical interactions, e.g., of KPNB1 with HSPA9; VCP with NCL; HNRNPA1 with HNRPF; KPNA2 with TXNIP; HNRPF with HNRPM; ACIN1 with SF3B4; and HNRPH1 with SFRS3, etc. In addition, 10 interactions were overlapped among the latency, LTNP and control condition, indicating that gene regulation was reprogrammed in different infection state.

A statistic analysis of latency and LTNP network topology was performed (**Fig. 3 B and C**). In latency network, the degree tended to decrease slowly complying with the power law y = ax^b^ where “a” is 36.310 and “b” is −0.786. The correlation coefficient was 0.285. In LTNP network, “a” is 876.97 and “b” is −2.139 with a correlation coefficient of 0.432. These values indicated a potential scale free future [23], and also suggested that the latency or LTNP network is assortative.

To validate if the network has small-world future, the duplication model in ‘RandomNetworks’ plugin of Cytoscape was used to construct random graphs [24], as it is a well-known model having power-law degree distributions and providing small-world networks [25]. We generated 10000 instances and computed the average clustering coefficient and degree distribution. The latency network exhibits dense local neighborhoods with an average clustering coefficient of 0.0495, which is higher than duplication model (0.0119±0.0637, p = 0.4438). The degree distribution in latency network is 6.3867, which is similar to that of random graphs generated by the duplication model (0.6203±3.3118, p = 0.9183), as expected for a small-world network [26]. The dense neighborhood feature of latency network suggested that it has modular organizations [27].

### Disease genes and hub identification

Recent studies using a large-scale siRNA screen identified over 600 host factors for HIV life cycle with 387 genes validated in at least two of three screens [28]–[32]. An overlap analysis of the DE genes found in our study with this dataset showed 23 coincident genes (Supplementary Table S2). These genes constitute the high confidence disease genes.

We calculated the significance of the degree difference between all the genes and disease genes in the network, and found that disease genes in latency setting have significantly higher degrees than all genes or those in control setting (**Fig. 4B**). We also found that the clustering coefficient of disease genes is significantly larger than that of all genes in the network (**Fig. 4C**). These results revealed that confident disease genes show increased local centrality in the network, and have more functional synergism to cause disease.

Since increased centrality is the key attribute of disease genes in the network, we calculated the putative hub genes present in our networks. In Table 1 were reported the top 10 hub genes obtained by hub analysis but only two of them (KPNA2 and ATP5G3) were selected as hubs by all the different algorithms in latency network. Centrality analysis also determined UGDH and CYFIP1 as hub nodes in LTNP network.

**Table 1:** Top 10 hub genes present in latency or LTNP network obtained by different algorithms and centrality measures.

MCC	MNC	EPC	Degree	Betweenness	Closeness	Stress
**Latency network**						
DDX11	DDX11	H2AFZ	H2AFZ	PSME3	DDX11	ATP5G3
F3	KPNA2	DDX11	DDX11	CFB	ATP5G3	H2AFZ
KPNA2	F3	ATP5G3	ATP5G3	ATP5G3	H2AFZ	PSME3
LILRB4	LILRB4	F3	KPNA2	CDC6	KPNA2	CFB
ATP5G3	SPOCK1	LILRB4	METTL1	KPNA2	PSME3	METTL1
IL8	IL8	KPNA2	CFB	METTL1	CDC6	KPNA2
SPOCK1	BMX	IL8	PSME3	H2AFZ	METTL1	CDC20
BMX	RBMX	METTL1	LILRB4	CDC20	F3	CDC6
H2AFZ	ATP5G3	PSME3	CDC6	RPS27	CFB	DDX11
METTL1	DAB2	RYR2	CDC20	ASPA	LILRB4	RPS27
**LTNP network**						
UGDH	UGDH	UGDH	UGDH	UGDH	UGDH	UGDH
CYFIP1	RBM28	CYFIP1	CYFIP1	CYFIP1	CYFIP1	CYFIP1
RBM28	PPFIBP1	CXCL6	MLSTD1	PIGQ	MLSTD1	CXCL6
PPFIBP1	STAT6	MLSTD1	MATN3	MLSTD1	CXCL6	YARS
CXCL6	STAMBPL1	MATN3	CXCL6	YARS	SLC1A6	PIGQ
STAT6	CYFIP1	SLC1A6	PIGQ	MATN3	YARS	MLSTD1
MLSTD1	PCAF	RBM28	YARS	CXCL6	MATN3	MATN3
MATN3	CLGN	STAT6	SLC1A6	SLC1A6	RBM28	SLC1A6
PIGQ	NEFH	PPFIBP1	RBM28	SERPINI1	ARL4C	ARL4C
YARS	TCN2	YARS	STAT6	ARL4C	PIGQ	RBM28

### Characterization of hub genes

It has been shown that high degree of connectivity correlates well with pleiotropic effects [33]. This indicated also that the most part of hub proteins in latency or LTNP network are involved in many different biological processes with different cellular localizations, more precisely KPNA2, and ATP5G3 are present in nucleus as well as cytoplasm whereas UGDH and CYFIP1 are in plasma membrane as well as cytoplasm.

**Figure 5:**
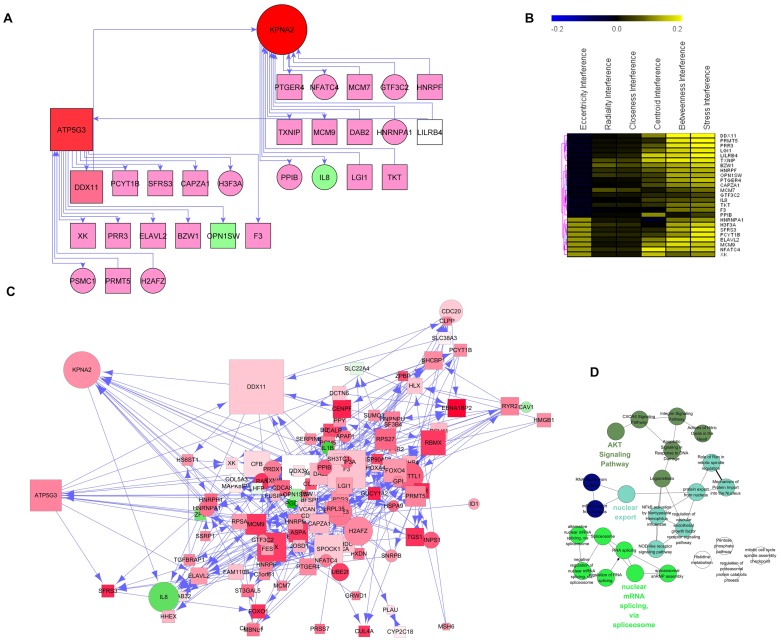
KPNA2 and ATP5G3 regulatory module. (**A**) KPNA2 and ATP5G3 first-order genes in the network. (**B**) When KPNA2 and ATP5G3 were both knocked out in the network, the positive interference genes were inferred by clustering analysis (pearson correlation). (**C**) KPNA2- ATP5G3 module, which include all the positive interference genes. (**D**) Gene ontology and pathway analysis of KPNA2- ATP5G3 module.

To evaluate the effect of hub gene as well as its associated regulatory elements, we reverse engineered the hub interactome by performing virtual knock-out experiments using interference method [34]. Topologically speaking, the hub genes take advantage (are positively influenced) by the presence in the network of the other related genes. As indicated in the **Figure 5B**, when KPNA2 and ATP5G3 were both knocked out, the most affected positive interferences genes constituted 3 clusters. First order of these genes in the latency network contained 123 genes. Gene ontology studies suggested that they are involved in important biological processes related to nuclear mRNA splicing, nuclear export and AKT signalling pathway.

**Figure 6:**
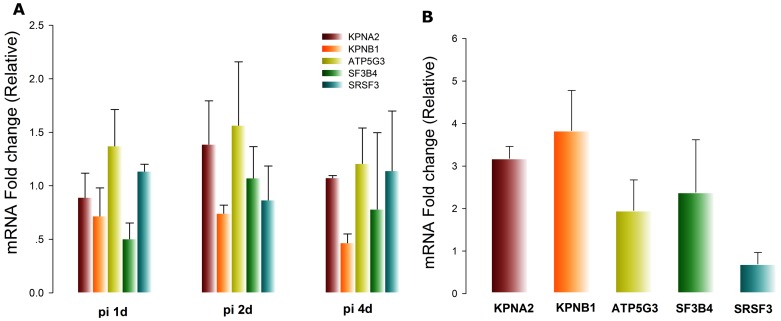
Dynamics of key genes during different infection mode. (**A**)After HIV-1 infection, real time PCR analysis of KPNA2, KPNB1, ATP5G3, SF3B4, and SRSF3 mRNA levels at the indicated time points post infection (pi). (**B**) mRNA levels of KPNA2, KPNB1, ATP5G3, SF3B4, and SRSF3 in HIV-1 latently infected cells. Data are representative of three experiments (average of three values ± standard deviation).

For the initial step to observe the dynamics of several these genes, CEM-SS cells were challenged with HIV-1 infection. At early infection stage (at 24 h post infection), genes contributing to the nuclear transport like KPNA2 and KPNB1, RNA splicing gene SF3B4 were down-regulated compared with the normal control. At later stage of HIV-1 life cycle, these genes as well as ATP5G3, showed a tendency of decreasing with the development of infection (**Fig. 6A**). An addition RNA splicing gene, SRSF3, which is not differential expressed between latency and LTNP groups, showed relatively steady across the infection. In contrast, in latently infected CEM-Bru cell line, KPNA2, KPNB1, ATP5G3, and SF3B4 were up-regulated 3.18±0.28, 3.84±0.94, 1.95±0.72, and 2.38±1.24 fold respectively when compared with the normal control (**Fig. 6B**). These results confirmed the DE genes from the microarray dataset analysis, and verified that these hub genes were up-regulated in the latently infected cells.

**Figure 7:**
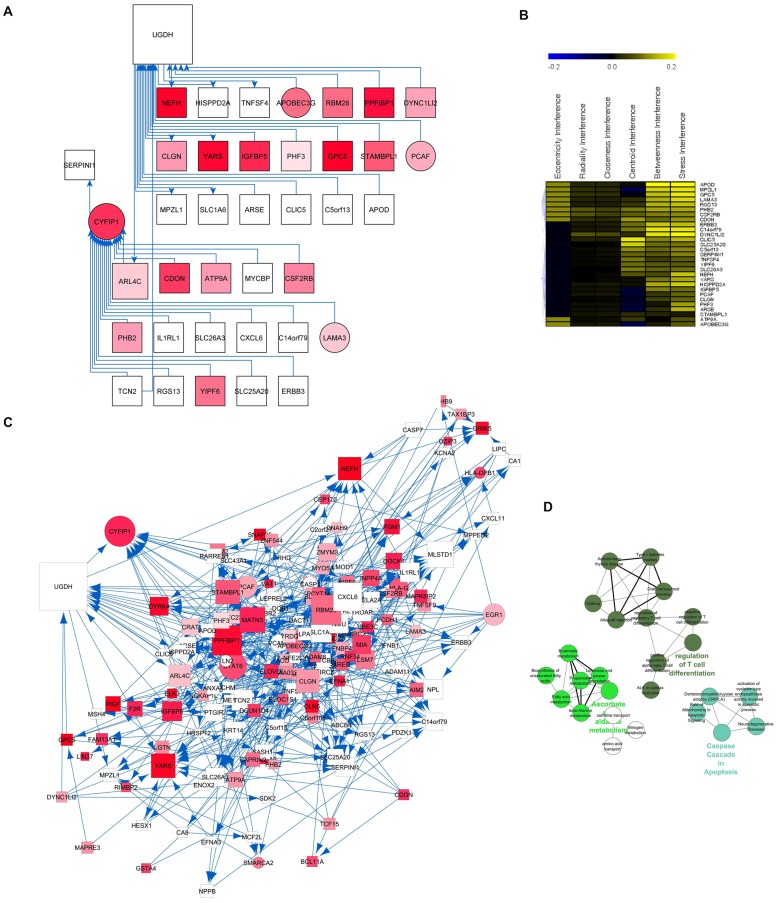
UGDH and CYFIP1 regulatory module. (**A**) UGDH and CYFIP1 first-order genes in the network. (**B**) The co-regulatory genes were inferred by knocking out UGDH and CYFIP1 in the network. (**C**) UGDH - CYFIP1 module, which include all the positive interference genes. (**D**) Gene ontology and pathway analysis of UGDH - CYFIP1 module.

In LTNP network, when UGDH and CYFIP1 were both knocked out, first order of the positive interference genes in the LTNP network contained 156 genes (**Fig. 7**). Gene ontology studies suggested that they are involved in biological processes related to regulation of T cell differentiation, caspase cascade in apoptosis and ascorbate metabolism.

## Discussion

By whole genome transcriptional profiling, molecular characterization of the LTNPs indicated that an up-regulation of components of MAPK, WNT, AKT and cytotoxic pathways contributing to cell survival and anti-viral responses [35], [36], and also up-regulation of genes related to cytokine–cytokine receptor interaction, actin cytoskeleton, and focal adhesion [37]. In contrast, up-regulated genes in progressors were mainly implicated in the regulation of DNA replication, cell cycle and DNA damage stimulus [37], or the pathways ranged from metabolism and energy production to mitochondria meditated cell apoptosis [36]. However, it is important to recognize that most previous works place emphasis on the comparison between HIV-1 LTNP and progressors.

A closer inspection of the differential expressed genes between HIV-1 latency and LTNP group in this study indicated that, a variety of well-established cofactors were identified in this study. 23 (5.044%) genes were supported by meta-analysis of HIV-1 replication associated genes [32], and 50 (5.622%) genes by HIV interaction database (Supplementary Table S2). The recovery of already implicated host factors was generally good in the overlap analysis, providing confidence about the authenticity of the newly called genes. Comparative function analysis of latency and LTNP group in this study clearly showed evidence for concerted up-regulation of nuclear transport, RNA metabolism, and AKT pathway in latency, and peroxisome proliferators or mitochondria in apoptosis signalling during LTNP state. Both group mediated focal adhesion, regulation of cell cycle transition, and antigen processing and presentation as well as immune response. It is well recognized that immunological or clinical properties of LTNP may be quite heterogeneous, and residual viral replication could be observed in the majority of LTNP patients using ultra-sensitive assays [38]. This study is consistent with the notion that residual viremia reflects release of virus from stable reservoirs rather than ongoing viral replication [1].

The analysis of gene regulatory network in specific disease context is an important step to define the biological process at the system biology level [23]. Due to the overlapping functions between latency and LTNP, to identify the molecular regulation of HIV latency in more detail, we assembled a gene regulation network based on the DE genes. Not only does the differential network predict many novel interactions, it also provides insights into the overall architecture of the regulation network in different infection states. For latency-enriched genes, we generated the network under latency, LTNP and control condition respectively. No significant difference of ever-known interactions were found among three states (P<0.05), and only 10 interactions were overlapped. These results indicated a reprogrammed regulatory tendency for specific disease condition.

Genes associated with a particular phenotype or function are not randomly positioned in the network, but tend to exhibit high connectivity that may cluster together and occur in central network locations [39]. Centrality tests demonstrated a significant difference between high-confidence disease genes and all genes under the certain condition (**Fig. 4**). These results validated the applicability of network based centrality test to rank disease genes.

Thus, in an attempt to understand and characterize the factors specifically associated with HIV-1 latency, we performed the hub analysis in the latency network, and found KPNA2 and ATP5G3 were the top two genes relevant to the latency regulation. KPNA2 is known as an adapter protein to mediate nuclear import and export in an energy requiring manner. Several studies found it stimulates pro-survival signal during stress response [40], and is natural resistance to infection [41]. ATP5G3 encodes a subunit of mitochondrial ATP synthase to catalyze ATP synthesis. Recent study suggested that genetic variants in nuclear-encoded mitochondrial genes influence AIDS progression [42]. knocking out KPNA2 and ATP5G3 in the network gained insight into the associated genes co-regulated in the network that constitute the synergized functional module.

At every step of the replication cycle, HIV-1 co-opts host proteins and cellular machineries to its advantage. The movement of proteins between the cytoplasm and nucleus mediated by the importins like KPNA2 is essential to many cellular processes such as differentiation and development, and also critical to HIV infection [43]. The KPNA2 -ATP5G3 module was closely associated with mitotic cell cycle, nuclear export, and nuclear mRNA splicing via spliceosome. These functions are in agreement with the previous study that the genes encoding proteasomes, nuclear transport factors, and splicing factors were up-regulated in latently infected cells [44]. KPNA2 -ATP5G3 module illustrated here raises the possibility that category nuclear export with contributing genes APAF1, DDX39A,HHEX,HNRNPA1, KPNA2,PXDN,RAN, and RNPS1,etc, category mRNA splicing with contributing genes HNRNPF,HNRNPH1,HNRNPM,HNRNPU, and TGS1,etc, category AKT signaling pathway with contributing genes APAF1,CAV1,FOXO1,FOXO4,IL1B,IL8,PIK3CA,PXDN,and RYR2, are the combined restriction factors for HIV replication. Supporting this speculation, several observations suggested that HIV infection enhances heterogeneous HNRNPA1 expression and promotes the relocalization of HNRNPA1 to the cytoplasm [45], which was dependent on the nuclear export of the unspliced viral RNA (vRNA). Depletion of HNRNPA increased expression of viral structural protein [46]. HNRNPH1 activates splicing of an HIV splicing substrate by promoting formation of ATP-dependent spliceosomal complexes [47]. Alteration in dosage of splicing factors was thought to diminish HIV replication by altering the ratios of the different HIV mRNA forms and the integration step [32]. At the nuclear transport step, recent study suggested that broad-spectrum inhibitor of importin α/β-mediated nuclear import like ivermectin has potent antiviral activity towards HIV [43]. Selective adjustment with the nuclear trafficking of proteins as a therapeutic strategy offers an attractive possibility to anti- HIV [48].

For LTNP network,UGDH and CYFIP1 were identified as the hubs. In details, UGDH is responsible for converting UDP-glucose to UDP-glucuronate and thereby participates in the biosynthesis of glycosaminoglycans such as hyaluronan, chondroitin sulfate, and heparan sulfate, which constitute the common components of the extracellular matrix (ECM) to mediate signal transduction and cell migration. CYFIP1 is a member of the actin-assembly-promoting Scar/WAVE complex, playing the roles in formation of membrane ruffles, lamellipodia, actin filament reorganization and axon outgrowth. Alterations in WAVE-regulated actin dynamics is correlated with impaired cell-cell adhesion and cell-ECM interactions [49]. Network analysis indicated that UGDH-CYFIP1 module in LTNP network mediates T cell differentiation, caspase cascade in apoptosis, and ascorbate and aldarate metabolism. It is implied that the main HIV limiting steps in LTNP is T cell differentiation and apoptosis control with the associated genes including HLA-DPB1, HLA-G,IFNB1,NCKAP1L,NPPB,TGFBR2, CASP1,CASP7,and SETX, etc. Recent studies identified that host cell microfilament cytoskeleton plays a wide range of roles in HIV infection, including viral entry, reverse transcription, transport to the nucleus, integration and finally a correct budding and release from the cell [50]. One explanation for non-progression could be there is a higher and better immunological synapsis between antigen specific CD^8+^ T cells and infected CD^4+^ T cells due to an adhesion processes and remodelling cytoskeleton taking place in the synapsis [51].

One limitation of this study is that all the available latency data sets used in this study are from transformed cell lines, which represent the traditional model for HIV latency [11]. Of note, in these models, there is no significant differences of expression ratios between zidovudine-treated latency cells and untreated cells, thus excluding the possibility that changes in gene expression were due to low levels of actively replicating viral population [44]. Due to the rare cells harboring latent virus *in vivo*, and the instability of using primary cells as models, there is still a need to establish the HIV latency model that best characterize the real features *in vivo*. However, large heterogeneous LTNP clinical samples used as background for comparison in this study could partially reflect the key points that describe the characteristics of HIV latency.

Though both HIV latency and LTNP showed virus replication restriction phenomenon, we used the gene regulatory network to differentiate these two pathogeneses. At this point, the dissimilar behavior of latency and LTNP might be due to the diverging mechanisms through differing network configuration. Besides the ever-known interactions of the networks, several novel function links in the network will be worthy of further experimental analysis.

In summary, by merging all the data available, our studies observed that a number of cellular genes and pathways are altered in viral latency that have not been previously associated with HIV infection, which may expand our knowledge of the factors involved in latency maintenance. Targeting KPNA2 associated co-factors may be particularly helpful in interfering with HIV latency, thus may provide new approaches to decrease or eliminate latent viral reservoirs.

## Materials and Methods

### Microarray data collection and merging

Previously, we have performed global transcription profile on a HIV latently infected CEM-SS cell (named CEM-Bru, GSE38634). We also selected datasets from the NCBI GEO database for available HIV latency or LTNP-related experiments (Latency: GSE1441, GSE1443; LTNP: GSE6740, GSE23879) and the corresponding normal control. Sample inclusion criteria were followed the original study [5], [44], [52]. The latency cells used in GSE1441 or GSE1443 were ACH-2 and U1, respectively. Ten LTNP samples were form GSE6740, and 12 elite controller samples from GSE23879. The data were generated with the different platforms that share a large number (5,917) of common genes.

Pre-processing were applied to microarray data to compute expression values in different experiments, according to the method suggested as previously described [5], [44], [52], which includes Robust Multi-array Average (RMA) and Quantile normalization. Systemic non-biological inter-laboratory experimental variation (‘batch effect’) between the datasets was adjusted using non-parametric empirical Bayes frameworks implemented in ComBat [16].

The quality of the merging dataset was assessed by principal variation component analysis (PVCA) and hierarchical clustering. PVCA method first reduces data dimension while maintaining the majority of the variability in the data, and then, variance component analysis (VCA) fits a mixed linear model using factors of interest as random (or batch) effects and other variables (or covariates) to estimate and partition the total variability [15]. Average linkage hierarchical cluster analysis was carried out using Mev software (http://www.tm4.org/mev/) with a pearson correlation as a similarity metric. The clustering was performed using all the genes in the platform.

### Differential expressed gene determination and function analysis

A linear model fit in conjunction with an empirical Bayes statistics were used to identify DE genes [53]. Adjustment for multiple testing was performed using the Benjamini-Hochberg adjustment. Candidate DE genes with fold change >2 and B-statistic >0 were used for the comparisons [36]. To identify the enriched functional categories from the DE genes, ClueGO was used to indentify significantly enriched gene-GO term or functional pathways [19]. ClueGO visualizes the selected terms in a functionally grouped annotation network that reflects the relationships between the terms based on the similarity of their associated genes using kappa statistics [19]. A double-sided hypergeometric test yielded the enrichment for GO-terms. Benjamini-Hochberg correction for multiple testing controlled the p-values.

### Network generation and clustering

Banjo (www.cs.duke.edu/~amink/software/banjo/) was used to infer the Bayesian network. The static Bayesian network inference algorithm was run based on the expression data by using standard parameters, with a discretization policy of q6. Consensus graphs, based on the top 100 networks, were obtained from at least 3×10^8^ searched networks. A file listing the parameter settings is provided as Supplementary data S1.

### Network topology analysis

Centrality parameters, such as betweenness centrality, closeness centrality and clustering efficient, etc, were analyzed by Networkanalyzer and Centiscape plugin of Cytoscape [24], [34]. Random-network plugin in Cytoscape was used to generate random networks. Maximal Clique Centrality (MCC), maximum neighborhood component (MNC), Edge Percolated component (EPC), and other centrality based measure were taken into account for exploring the gene essentiality (hub) in network.

### Cells, viral infection and quantitative real-time PCR

CEM-Bru is a latently infected cell line harboring the HIV-1 Bru strain by limit dilution cloning process, while CEM-SS is the corresponding parental uninfected cell line. All cells were cultured with Dulbecco's modified Eagle's medium (DMEM) supplemented with 2 mM L-glutamine, 5% penicillin-streptomycin and 10% fetal calf serum at 37°C in a humidified atmosphere (5% CO_2_). HIV-1 Lai stock was produced on H9/IIIB cells, and the virus-containing supernatant was filter, frozen in aliquots at −80°C. CEM-SS cells were seeded at 1×10^6^ cells/ml and treated with HIV-1 Lai at a multiplicity of infection (MOI) of 2 for 2 h. Total RNA was isolated using the Trizol method (Invitrogen) as suggested by the manufacturer. 1 µg of total RNA was reverse-transcribed using the PrimeScript™ Reverse Transcriptase (Takara) in 20 µl total volume using random hexamers. Real time PCR was performed using the SYBR® Premix Ex Taq kit (Takara) and 0.2 µM of gene specific primers (Supplementary Table S3). All reactions were run in the Opticon II Real-Time PCR System (BioRad). Melting curve analysis allowed testing for specificity of the amplicon. The relative quantification expression was calculated using the delta-delta Ct method with each gene normalized to GAPDH.

## Supporting Information

Data S1 **Parameter settings used in Banjo to infer gene regulatory network.**
(TXT)

Table S1 **Differential expressed genes between HIV-1 latency and LTNP.**
(PDF)

Table S2 **Overlap analysis of the DE genes found in our study with other data set.**
(PDF)

Table S3 **Primers used in this study.**
(PDF)

Figure S1 **The network of latency over-expressed genes in LTNP state.**
(PDF)

Figure S2 **The network of latency over-expressed genes in normal control state.**
(PDF)

Figure S3 **The network of LTNP over-expressed genes in latency state.**
(PDF)

Figure S4 **The network of LTNP over-expressed genes in normal control state.**
(PDF)
