# Supplementary material for: Bridging HIV-1 Cellular Latency and Clinical Long-Term Non-Progressor: An Interactomic View
Source: PLoS One. 2013 Feb 25;8(2):e55791. doi: 10.1371/journal.pone.0055791 (PMC3581534; doi:10.1371/journal.pone.0055791)
Supplement: Table S1 — Differential expressed genes between HIV-1 latency and LTNP. (PDF) [file pone.0055791.s002.pdf]

SUPPLEMENTARY DATA

TABLES

TableS1 Differential expressed genes between HIV-1 latency and LTNP

Gene symbol	Gene ID	Log fold change	Fold change	Adjust p values[a]
**Latency over-expressed genes**				
**USP9Y**	8287	2.476	5.564	1.312E-06
**DDX3Y**	8653	2.398	5.271	1.953E-07
**FES**	2242	2.365	5.152	2.877E-14
**GUCY2C**	2984	2.255	4.773	5.659E-17
**MAPK8IP1**	9479	2.154	4.452	1.520E-10
**ZFY**	7544	2.148	4.431	2.383E-06
**FOXO1**	2308	2.082	4.233	3.919E-11
**SFRS3**	6428	2.065	4.183	5.583E-24
**GUCY1A2**	2977	2.036	4.100	4.095E-11
**TGS1**	96764	2.027	4.075	4.901E-17
**KPNB1**	3837	2.001	4.004	3.219E-18
**EBNA1BP2**	10969	1.950	3.865	4.095E-09
**CCNE1**	898	1.944	3.848	1.197E-11
**TSPYL5**	85453	1.910	3.759	6.276E-20
**PPIF**	10105	1.884	3.692	6.979E-16
**CORO2B**	10391	1.871	3.657	2.213E-11
**ASPA**	443	1.868	3.651	1.822E-13
**ACR**	49	1.868	3.650	1.076E-20
**SERPINB2**	5055	1.867	3.649	1.069E-06
**HSP90AB1**	3326	1.831	3.559	6.429E-11
**MCM9**	254394	1.825	3.543	1.574E-16
**RBMX**	27316	1.820	3.531	2.277E-24
**TNFRSF21**	27242	1.792	3.464	4.172E-08
**RAN**	5901	1.786	3.448	4.748E-16
**SCN4A**	6329	1.764	3.397	4.510E-13
**CFB**	629	1.742	3.345	1.002E-09
**CUL4A**	8451	1.738	3.335	2.026E-18
**ELAVL2**	1993	1.680	3.204	1.371E-11
**CNTN2**	6900	1.675	3.192	1.048E-21
**UBE2I**	7329	1.670	3.182	1.092E-11
**DDX39**	10212	1.649	3.136	9.149E-17
**SLC22A4**	6583	1.646	3.129	1.604E-07
**PPIB**	5479	1.637	3.110	1.461E-10
**METTL1**	4234	1.630	3.095	2.582E-12
**BMX**	660	1.628	3.091	1.490E-10
**XK**	7504	1.620	3.075	3.528E-10
**RBM3**	5935	1.616	3.066	4.995E-14
**ZPBP**	11055	1.610	3.053	8.150E-10

**HSPA9**	3313	1.600	3.032	1.540E-18
**SFRP1**	6422	1.595	3.020	1.131E-06
**ACTB**	60	1.583	2.996	4.641E-17
**PXDN**	7837	1.576	2.982	2.455E-09
**CALR**	811	1.568	2.964	1.461E-10
**NOL5A**	10528	1.546	2.921	3.955E-11
**NFATC4**	4776	1.545	2.919	7.227E-09
**CYP1B1**	1545	1.536	2.900	1.635E-06
**KERA**	11081	1.526	2.880	1.089E-13
**RNPS1**	10921	1.520	2.868	1.153E-11
**EPHB4**	2050	1.519	2.866	2.710E-07
**CCT7**	10574	1.505	2.838	2.582E-12
**ID1**	3397	1.498	2.825	1.498E-13
**SR140**	23350	1.497	2.822	6.014E-15
**PCYT1B**	9468	1.488	2.804	1.129E-13
**EIF3G**	8666	1.474	2.778	3.992E-17
**CDC20**	991	1.470	2.770	2.817E-09
**FUS**	2521	1.466	2.763	1.988E-13
**SUMO1**	7341	1.462	2.755	8.858E-14
**CASQ2**	845	1.444	2.720	1.688E-07
**SERPINB6**	5269	1.443	2.719	3.765E-05
**ST3GAL5**	8869	1.418	2.672	4.018E-09
**TXNIP**	10628	1.412	2.662	2.721E-14
**LIMCH1**	22998	1.412	2.660	1.341E-06
**HNRPH1**	3187	1.409	2.656	8.722E-13
**SRF**	6722	1.405	2.648	3.690E-19
**ATP1A2**	477	1.399	2.637	2.717E-15
**C1S**	716	1.388	2.617	1.870E-09
**PPY**	5539	1.387	2.615	3.712E-07
**BZW1**	9689	1.382	2.607	3.028E-12
**C12orf4**	57102	1.380	2.602	1.871E-08
**PLAU**	5328	1.373	2.591	4.498E-07
**GCKR**	2646	1.363	2.573	8.878E-09
**BAG1**	573	1.363	2.571	3.128E-07
**CDC6**	990	1.361	2.569	5.118E-06
**CAPZA1**	829	1.358	2.564	1.788E-05
**RPL35**	11224	1.354	2.557	2.486E-17
**CTSW**	1521	1.340	2.532	1.075E-08
**BACH1**	571	1.338	2.529	6.700E-10
**CYP17A1**	1586	1.331	2.516	3.363E-07
**C1orf61**	10485	1.325	2.505	1.875E-09
**RPS27**	6232	1.323	2.501	3.038E-11
**NCL**	4691	1.318	2.493	5.240E-11
**H3F3A**	3020	1.318	2.493	1.638E-11

**IL1B**	3553	1.317	2.492	4.318E-05
**CDCA8**	55143	1.298	2.458	3.936E-09
**GRWD1**	83743	1.294	2.452	9.233E-14
**PTGER4**	5734	1.294	2.452	2.448E-06
**IL8**	3576	1.293	2.451	4.248E-06
**ETF1**	2107	1.290	2.445	1.039E-13
**TRPC3**	7222	1.288	2.441	9.715E-06
**APEX1**	328	1.281	2.430	1.063E-09
**ZNF200**	7752	1.275	2.420	2.426E-12
**CENPF**	1063	1.271	2.413	1.854E-04
**ADRBK1**	156	1.269	2.410	8.257E-06
**HS6ST1**	9394	1.264	2.402	2.951E-11
**HHEX**	3087	1.261	2.397	1.261E-07
**PCTK1**	5127	1.252	2.382	4.234E-07
**KPNA2**	3838	1.242	2.365	7.264E-12
**RRS1**	23212	1.234	2.353	3.299E-06
**GFPT2**	9945	1.234	2.352	4.037E-04
**HSP90B1**	7184	1.232	2.349	3.702E-08
**PRKD1**	5587	1.223	2.334	1.365E-05
**BFSP1**	631	1.221	2.331	1.408E-07
**PGC**	5225	1.219	2.327	8.720E-05
**ENTPD2**	954	1.211	2.316	3.363E-07
**LRP1**	4035	1.211	2.315	4.429E-13
**RAB32**	10981	1.210	2.314	1.380E-06
**SUMO3**	6612	1.208	2.309	5.414E-16
**CHST10**	9486	1.206	2.307	7.309E-12
**LPPR4**	9890	1.205	2.306	2.987E-11
**DCTN6**	10671	1.204	2.303	1.825E-11
**F3**	2152	1.202	2.300	2.204E-06
**FTL**	2512	1.201	2.300	4.231E-10
**SF3B4**	10262	1.198	2.294	6.550E-14
**RHBDL1**	9028	1.198	2.294	1.154E-07
**CYP2C18**	1562	1.193	2.286	5.079E-06
**BAMBI**	25805	1.188	2.278	2.847E-11
**CLPP**	8192	1.183	2.271	9.592E-07
**PIK3CA**	5290	1.183	2.271	7.445E-08
**PGM5**	5239	1.183	2.270	1.350E-08
**SRM**	6723	1.182	2.268	6.054E-07
**LHFP**	10186	1.179	2.265	3.765E-05
**HNRNPA1**	3178	1.179	2.264	6.302E-09
**OPN1SW**	611	1.174	2.256	3.551E-05
**TGFBRAP1**	9392	1.171	2.251	1.604E-07
**GPI**	2821	1.168	2.247	5.318E-10
**ATP6V0B**	533	1.167	2.245	1.688E-15

**FEN1**	2237	1.166	2.243	4.036E-10
**SLC6A7**	6534	1.163	2.240	7.451E-07
**RAMP2**	10266	1.163	2.238	2.333E-11
**HDC**	3067	1.162	2.237	7.162E-05
**HLX**	3142	1.160	2.234	1.778E-07
**ACIN1**	22985	1.156	2.228	2.527E-16
**APAF1**	317	1.151	2.221	4.091E-07
**COL5A3**	50509	1.149	2.217	8.144E-06
**ATP5H**	10476	1.146	2.213	4.271E-06
**LGI1**	9211	1.144	2.210	1.067E-06
**PPP1R2**	5504	1.141	2.205	6.609E-18
**HNRPUL1**	11100	1.139	2.203	9.229E-13
**SPOCK1**	6695	1.137	2.199	2.945E-04
**ACTG1**	71	1.134	2.194	8.823E-08
**PPRC1**	23082	1.129	2.187	6.274E-06
**VCAN**	1462	1.128	2.185	8.709E-09
**FAM110B**	90362	1.127	2.184	4.782E-08
**MAGOH**	4116	1.125	2.181	4.251E-11
**PER1**	5187	1.121	2.175	1.258E-04
**HOXA4**	3201	1.109	2.157	6.559E-07
**PRR3**	80742	1.107	2.154	2.661E-12
**SH3TC1**	54436	1.104	2.150	1.894E-09
**PSMC1**	5700	1.099	2.141	5.790E-15
**NOL1**	4839	1.096	2.138	7.016E-11
**HNRNPU**	3192	1.093	2.133	5.240E-11
**FUSIP1**	10772	1.092	2.132	3.648E-12
**EVI1**	2122	1.091	2.131	3.431E-06
**SHCBP1**	79801	1.086	2.123	2.525E-08
**DDX11**	1663	1.083	2.118	7.222E-09
**HNRNPC**	3183	1.082	2.116	1.772E-11
**EIF4A1**	1973	1.080	2.114	1.421E-16
**MCM7**	4176	1.078	2.111	3.448E-07
**CLCA1**	1179	1.076	2.108	3.344E-07
**PSME3**	10197	1.074	2.105	1.342E-06
**ARHGDIB**	397	1.073	2.104	1.808E-08
**TCEB2**	6923	1.071	2.100	2.540E-05
**HNRPF**	3185	1.070	2.100	4.330E-08
**SF3A3**	10946	1.070	2.100	1.902E-07
**KIF23**	9493	1.069	2.098	1.862E-06
**BHLHB3**	79365	1.069	2.097	7.172E-07
**JOSD1**	9929	1.067	2.095	1.004E-12
**CD200**	4345	1.066	2.094	2.885E-07
**CHD1**	1105	1.066	2.094	2.058E-09
**FOXO4**	4303	1.066	2.093	4.055E-11

**RASSF1**	11186	1.065	2.093	7.455E-06
**HMGB1**	3146	1.063	2.089	1.612E-13
**TOR1AIP1**	26092	1.060	2.085	5.338E-09
**HNRPM**	4670	1.059	2.084	1.152E-05
**RYR2**	6262	1.059	2.083	3.102E-05
**EDNRA**	1909	1.059	2.083	4.430E-06
**CAV1**	857	1.058	2.082	1.661E-04
**CDH4**	1002	1.057	2.080	5.405E-06
**PRSS7**	5651	1.056	2.079	1.463E-09
**SNRPB**	6628	1.054	2.076	2.427E-08
**POP7**	10248	1.054	2.076	7.746E-13
**ARHGAP19**	84986	1.052	2.074	5.986E-06
**UTP14A**	10813	1.050	2.071	1.037E-08
**TKT**	7086	1.038	2.053	2.511E-09
**NDUFV2**	4729	1.037	2.052	2.172E-09
**MBNL1**	4154	1.037	2.052	6.678E-07
**ATP5G3**	518	1.036	2.051	1.723E-07
**H2AFZ**	3015	1.033	2.047	8.738E-10
**CHRNA1**	1134	1.032	2.044	1.351E-04
**VCP**	7415	1.031	2.043	7.731E-08
**RPSA**	3921	1.030	2.043	1.350E-08
**ARSJ**	79642	1.030	2.042	5.244E-04
**GTF3C2**	2976	1.030	2.042	1.611E-07
**PRMT5**	10419	1.028	2.039	9.568E-09
**DNPEP**	23549	1.025	2.035	7.930E-13
**GEMIN7**	79760	1.024	2.033	1.017E-07
**AKAP6**	9472	1.024	2.033	3.552E-05
**ZFAND6**	54469	1.023	2.032	5.886E-06
**DCLK1**	9201	1.022	2.031	1.657E-07
**MSH6**	2956	1.021	2.029	1.797E-09
**SLC38A3**	10991	1.020	2.028	4.018E-05
**PSMD6**	9861	1.017	2.023	1.397E-10
**AAMP**	14	1.016	2.022	4.055E-11
**SLC7A11**	23657	1.015	2.021	1.561E-04
**SSRP1**	6749	1.013	2.019	6.888E-08
**DAB2**	1601	1.009	2.012	3.392E-04
**PRDX1**	5052	1.007	2.010	6.364E-07
**C9orf61**	9413	1.006	2.008	2.285E-05
**DNAJC7**	7266	1.006	2.008	2.153E-11
**LILRB4**	11006	1.004	2.005	3.186E-04
**IL23A**	51561	1.001	2.002	6.139E-05
**BCL6**	604	1.001	2.001	5.739E-10
**Latency under-expressed genes**				

**MAPRE3**	22924	-1.001	-2.001	1.523E-09
**CXCL6**	6372	-1.001	-2.001	7.970E-05
**TCF15**	6939	-1.001	-2.002	1.596E-04
**LIPC**	3990	-1.002	-2.002	2.638E-05
**CCND1**	595	-1.002	-2.002	1.805E-12
**TRPA1**	8989	-1.004	-2.005	8.963E-05
**ZMYM3**	9203	-1.006	-2.008	7.982E-13
**CLIC5**	53405	-1.008	-2.012	1.834E-06
**CAPN3**	825	-1.010	-2.014	6.547E-09
**RHD**	6007	-1.011	-2.016	2.400E-05
**CDON**	50937	-1.013	-2.018	1.878E-06
**AREG**	374	-1.014	-2.019	1.586E-05
**LGTN**	1939	-1.015	-2.020	1.029E-14
**SPOCK3**	50859	-1.016	-2.023	7.977E-06
**PHB2**	11331	-1.019	-2.027	5.537E-08
**CD33**	945	-1.022	-2.030	4.791E-04
**JRKL**	8690	-1.022	-2.030	1.173E-10
**MAPK8IP2**	23542	-1.025	-2.035	3.781E-05
**N4BP2L2**	10443	-1.027	-2.037	1.343E-13
**TOR1B**	27348	-1.029	-2.040	1.251E-05
**PHTF2**	57157	-1.029	-2.040	3.585E-09
**LGALS3BP**	3959	-1.031	-2.043	1.598E-09
**NME5**	8382	-1.033	-2.046	7.816E-05
**GOLPH3L**	55204	-1.034	-2.048	4.894E-09
**ENDOG**	2021	-1.036	-2.050	4.724E-06
**ZNF544**	27300	-1.037	-2.052	5.850E-07
**YIPF6**	286451	-1.040	-2.056	4.894E-09
**EPHB6**	2051	-1.042	-2.059	1.770E-05
**SNRPF**	6636	-1.042	-2.059	2.686E-10
**NFE2L3**	9603	-1.042	-2.059	6.655E-11
**CHM**	1121	-1.044	-2.062	3.631E-10
**BCL11A**	53335	-1.044	-2.063	1.125E-06
**EDEM2**	55741	-1.045	-2.063	1.732E-09
**EPHX2**	2053	-1.045	-2.063	1.185E-05
**FNBP4**	23360	-1.046	-2.065	1.871E-08
**CAPRIN2**	65981	-1.048	-2.067	9.233E-14
**LAMA3**	3909	-1.049	-2.069	9.947E-07
**TGFBR2**	7048	-1.050	-2.070	4.815E-07
**UBE3C**	9690	-1.050	-2.071	4.573E-09
**GPC5**	2262	-1.052	-2.073	1.653E-05
**TFAP2B**	7021	-1.053	-2.075	3.188E-07
**GSTA4**	2941	-1.054	-2.077	5.584E-07
**DNTT**	1791	-1.056	-2.079	1.759E-05
**TMPRSS2**	7113	-1.058	-2.082	1.181E-07

**DOCK1**	1793	-1.060	-2.085	1.464E-04
**CLN5**	1203	-1.066	-2.094	1.995E-13
**DENND2D**	79961	-1.074	-2.105	1.389E-07
**ADCY7**	113	-1.079	-2.113	3.852E-13
**STAMBPL1**	57559	-1.080	-2.114	1.615E-08
**DYNC1LI2**	1783	-1.080	-2.114	1.590E-09
**ACOX3**	8310	-1.082	-2.117	1.343E-11
**AVPR2**	554	-1.083	-2.118	3.619E-05
**UBL4A**	8266	-1.083	-2.119	9.668E-11
**DCUN1D4**	23142	-1.084	-2.119	8.699E-11
**C2orf27**	29798	-1.085	-2.121	1.004E-04
**C5orf13**	9315	-1.085	-2.121	6.591E-07
**DZIP3**	9666	-1.087	-2.124	2.648E-05
**ELOVL6**	79071	-1.087	-2.125	5.705E-06
**ELA2A**	63036	-1.087	-2.125	5.966E-07
**EGR1**	1958	-1.089	-2.128	3.556E-05
**TADA2L**	6871	-1.090	-2.128	3.966E-08
**ADAM11**	4185	-1.093	-2.133	2.138E-06
**BUB1B**	701	-1.093	-2.133	1.360E-07
**KDR**	3791	-1.094	-2.134	1.362E-07
**C11orf60**	56912	-1.094	-2.135	3.069E-08
**BLOC1S1**	2647	-1.095	-2.136	1.520E-10
**PPFIBP1**	8496	-1.098	-2.140	8.484E-10
**PSMB3**	5691	-1.100	-2.143	9.363E-08
**PHEX**	5251	-1.100	-2.143	4.749E-05
**PIGF**	5281	-1.106	-2.152	1.765E-14
**NPC2**	10577	-1.107	-2.153	4.492E-05
**DAO**	1610	-1.108	-2.155	5.277E-06
**PRKAR2B**	5577	-1.111	-2.160	8.503E-06
**RARRES1**	5918	-1.112	-2.162	5.378E-08
**FOXN2**	3344	-1.114	-2.164	1.282E-08
**TCN2**	6948	-1.119	-2.172	8.747E-05
**APOBEC3G**	60489	-1.121	-2.175	1.881E-08
**MATN3**	4148	-1.121	-2.175	1.877E-08
**SPOCK2**	9806	-1.126	-2.182	2.560E-09
**ARSE**	415	-1.126	-2.183	4.566E-06
**CASP5**	838	-1.128	-2.186	1.281E-06
**IGFBP5**	3488	-1.136	-2.198	5.061E-06
**GGT1**	2678	-1.139	-2.202	2.144E-09
**CASP7**	840	-1.141	-2.206	6.032E-08
**C6orf105**	84830	-1.142	-2.206	1.252E-12
**KIAA0329**	9895	-1.143	-2.208	2.323E-04
**PEPD**	5184	-1.143	-2.209	1.918E-14
**MYCBP**	26292	-1.144	-2.209	2.931E-13

**GTF2E2**	2961	-1.147	-2.215	3.709E-15
**PCAF**	8850	-1.148	-2.217	1.311E-04
**KLK10**	5655	-1.149	-2.217	1.260E-07
**TROAP**	10024	-1.149	-2.217	2.361E-05
**BIRC3**	330	-1.149	-2.218	1.118E-04
**INPP4A**	3631	-1.150	-2.219	1.409E-09
**CEP68**	23177	-1.150	-2.220	6.757E-12
**CXADR**	1525	-1.160	-2.235	1.216E-06
**MB**	4151	-1.163	-2.240	9.473E-06
**HESX1**	8820	-1.168	-2.247	3.030E-06
**ERG**	2078	-1.168	-2.247	2.517E-07
**KRT14**	3861	-1.177	-2.261	8.503E-06
**TNFSF9**	8744	-1.182	-2.269	7.605E-07
**CSF2RB**	1439	-1.182	-2.269	3.647E-04
**CA8**	767	-1.183	-2.270	6.509E-10
**IFNB1**	3456	-1.183	-2.271	2.494E-04
**MYT1**	4661	-1.189	-2.280	8.821E-06
**GLCE**	26035	-1.191	-2.283	2.840E-09
**NUTF2**	10204	-1.197	-2.292	2.037E-07
**CRAT**	1384	-1.199	-2.295	8.019E-08
**SEC23A**	10484	-1.201	-2.299	2.731E-13
**FABP1**	2168	-1.204	-2.303	1.817E-07
**PEX3**	8504	-1.206	-2.306	1.563E-12
**MYO5A**	4644	-1.207	-2.308	1.058E-09
**NPPB**	4879	-1.207	-2.309	2.034E-07
**LEPREL2**	10536	-1.210	-2.314	4.455E-11
**LPA**	4018	-1.212	-2.317	6.192E-08
**TJP2**	9414	-1.213	-2.318	7.038E-11
**TLN2**	83660	-1.214	-2.320	1.494E-06
**ANXA4**	307	-1.216	-2.323	1.935E-06
**MIA**	8190	-1.219	-2.328	1.709E-09
**IGFBP3**	3486	-1.219	-2.328	5.431E-08
**PCYT1A**	5130	-1.223	-2.334	8.851E-16
**RBM28**	55131	-1.223	-2.335	2.121E-09
**BHLHB9**	80823	-1.228	-2.343	1.213E-10
**TAP1**	6890	-1.233	-2.350	1.546E-14
**SNAP25**	6616	-1.234	-2.352	7.027E-10
**KCNA2**	3737	-1.238	-2.358	1.188E-09
**ANXA3**	306	-1.244	-2.369	2.122E-06
**BRCA1**	672	-1.249	-2.377	2.062E-10
**F2R**	2149	-1.251	-2.380	1.594E-06
**HRSP12**	10247	-1.253	-2.383	3.538E-12
**IL6R**	3570	-1.253	-2.384	5.969E-10
**ELA2**	1991	-1.255	-2.387	3.411E-06

**GFAP**	2670	-1.256	-2.388	3.741E-07
**PPAP2C**	8612	-1.256	-2.388	9.334E-09
**AKT1**	207	-1.257	-2.389	6.510E-08
**ADAM8**	101	-1.257	-2.390	5.829E-12
**PRIM1**	5557	-1.259	-2.393	9.307E-10
**BLVRB**	645	-1.260	-2.395	8.278E-16
**STAT6**	6778	-1.260	-2.395	8.433E-24
**TST**	7263	-1.261	-2.397	1.154E-07
**PHF3**	23469	-1.264	-2.402	1.498E-15
**VBP1**	7411	-1.266	-2.404	1.418E-14
**UBAC1**	10422	-1.266	-2.404	2.956E-10
**TM4SF5**	9032	-1.267	-2.407	6.953E-07
**ME1**	4199	-1.269	-2.410	1.039E-07
**DYRK4**	8798	-1.272	-2.415	2.723E-13
**KRT16**	3868	-1.276	-2.422	2.042E-06
**PPFIA4**	8497	-1.286	-2.438	6.890E-07
**APOD**	347	-1.287	-2.440	3.969E-08
**RGS13**	6003	-1.294	-2.453	2.324E-05
**DGKI**	9162	-1.295	-2.453	1.342E-06
**ABCB4**	5244	-1.299	-2.461	2.571E-09
**ALDH3A2**	224	-1.301	-2.463	1.361E-11
**EFNA1**	1942	-1.306	-2.473	5.829E-12
**CXCL11**	6373	-1.308	-2.476	1.416E-10
**HLA-G**	3135	-1.316	-2.489	3.913E-17
**MPP3**	4356	-1.326	-2.507	1.403E-06
**DACT1**	51339	-1.326	-2.507	1.580E-06
**ERBB3**	2065	-1.328	-2.510	2.155E-10
**ARHGEF12**	23365	-1.330	-2.514	1.002E-09
**LMOD1**	25802	-1.335	-2.523	2.580E-07
**SLC25A20**	788	-1.336	-2.525	1.955E-07
**RIMBP2**	23504	-1.338	-2.528	1.399E-07
**TAX1BP3**	30851	-1.343	-2.536	1.116E-12
**KL**	9365	-1.347	-2.544	1.703E-06
**PCDH1**	5097	-1.350	-2.549	1.326E-07
**CEP170**	9859	-1.352	-2.553	1.818E-15
**UGDH**	7358	-1.359	-2.564	8.722E-13
**MPPED2**	744	-1.371	-2.586	2.848E-06
**PIGQ**	9091	-1.372	-2.589	1.015E-11
**BDH2**	56898	-1.378	-2.598	1.805E-12
**HISPPD2A**	9677	-1.386	-2.614	1.988E-13
**C14orf79**	122616	-1.395	-2.630	2.078E-08
**AIM2**	9447	-1.397	-2.633	2.058E-09
**SMARCA2**	6595	-1.398	-2.636	1.288E-18
**ATP9A**	10079	-1.403	-2.645	1.992E-12

**STT3A**	3703	-1.413	-2.663	1.716E-10
**LSM7**	51690	-1.414	-2.666	9.149E-17
**NPL**	80896	-1.416	-2.669	4.578E-07
**SLC1A6**	6511	-1.418	-2.672	1.437E-08
**ENO2**	2026	-1.419	-2.673	5.908E-07
**ISG15**	9636	-1.423	-2.681	6.013E-08
**CUL7**	9820	-1.430	-2.694	8.193E-18
**MCF2L**	23263	-1.431	-2.696	3.702E-08
**SLC26A3**	1811	-1.434	-2.702	6.723E-08
**ARL4C**	10123	-1.453	-2.739	2.670E-10
**TNFSF4**	7292	-1.462	-2.754	8.453E-09
**IL1RL1**	9173	-1.463	-2.757	4.169E-10
**DNAH9**	1770	-1.473	-2.776	3.992E-11
**SDK2**	54549	-1.497	-2.822	3.462E-08
**PEO1**	56652	-1.498	-2.824	3.511E-11
**NEFH**	4744	-1.498	-2.825	1.266E-10
**C8B**	732	-1.501	-2.831	6.979E-09
**ZNF189**	7743	-1.508	-2.845	4.302E-19
**STC2**	8614	-1.509	-2.846	3.560E-08
**LIN37**	55957	-1.517	-2.862	2.353E-23
**NCKAP1L**	3071	-1.518	-2.864	2.277E-24
**CLGN**	1047	-1.519	-2.867	4.248E-16
**PRICKLE3**	4007	-1.534	-2.895	6.231E-08
**SETX**	23064	-1.535	-2.899	4.390E-25
**ELK3**	2004	-1.545	-2.919	2.547E-11
**TGFA**	7039	-1.559	-2.947	1.037E-10
**MPZL1**	9019	-1.560	-2.949	3.943E-16
**TEX10**	54881	-1.589	-3.009	6.427E-20
**PALM**	5064	-1.602	-3.036	4.803E-11
**KIAA0802**	23255	-1.612	-3.057	1.374E-08
**VCAM1**	7412	-1.621	-3.075	3.567E-06
**VLDLR**	7436	-1.631	-3.098	1.486E-10
**VAT1**	10493	-1.657	-3.154	5.379E-14
**HLA-DPB1**	3115	-1.657	-3.154	2.052E-09
**HLA-DQB1**	3119	-1.666	-3.173	1.096E-07
**CASP1**	834	-1.671	-3.184	2.486E-17
**NMU**	10874	-1.680	-3.204	2.790E-08
**ENOX2**	10495	-1.682	-3.209	1.805E-12
**ADAM10**	102	-1.683	-3.210	2.516E-16
**PGM1**	5236	-1.683	-3.211	1.617E-15
**FZD1**	8321	-1.683	-3.212	1.953E-07
**CA1**	759	-1.683	-3.212	3.313E-10
**YRDC**	79693	-1.694	-3.237	4.207E-09
**ADAMTS3**	9508	-1.697	-3.243	6.293E-09

**EFNA3**	1944	-1.708	-3.266	1.505E-09
**YEATS2**	55689	-1.711	-3.273	1.127E-17
**MSH4**	4438	-1.711	-3.273	5.066E-14
**MLSTD1**	55711	-1.723	-3.300	1.429E-08
**NIPSNAP1**	8508	-1.725	-3.305	2.197E-27
**SASH1**	23328	-1.727	-3.310	1.977E-15
**YARS**	8565	-1.775	-3.423	4.414E-11
**LOC51149**	51149	-1.793	-3.465	2.513E-13
**CYFIP1**	23191	-1.797	-3.476	2.023E-22
**PDZK1**	5174	-1.858	-3.625	1.612E-13
**BHLHB2**	8553	-1.936	-3.827	1.808E-08
**GRIK5**	2901	-1.954	-3.875	2.007E-23
**RNF34**	80196	-1.969	-3.914	2.197E-27
**GRK4**	2868	-1.991	-3.976	1.469E-19
**SERPINI1**	5274	-2.027	-4.074	8.092E-09
**PHACTR2**	9749	-2.044	-4.125	4.347E-20
**RPRM**	56475	-2.094	-4.269	2.717E-10
**FGD1**	2245	-2.138	-4.400	4.757E-12
**PTGIR**	5739	-2.176	-4.520	3.281E-14
**FAM13A1**	10144	-2.180	-4.530	1.533E-10
**SLC43A1**	8501	-2.287	-4.879	4.070E-25
**BIRC2**	329	-2.380	-5.204	2.189E-30
**EHHADH**	1962	-2.395	-5.260	1.796E-14
**SPTLC3**	55304	-2.524	-5.750	1.874E-18

[a] Benjamini-Hochberg correction for multiple testing controlled the P-values
