# Supplementary material for: Bridging HIV-1 Cellular Latency and Clinical Long-Term Non-Progressor: An Interactomic View
Source: PLoS One. 2013 Feb 25;8(2):e55791. doi: 10.1371/journal.pone.0055791 (PMC3581534; doi:10.1371/journal.pone.0055791)
Supplement: Table S2 — Overlap analysis of the DE genes found in our study with other data set. (PDF) [file pone.0055791.s003.pdf]

SUPPLEMENTARY DATA

TABLES

TableS2 Differential expressed genes overlapping with ever-known HIV infection co-factors

DE genes overlapping with SiRNA dataset[a]	Gene symbol	Gene ID	Regulation
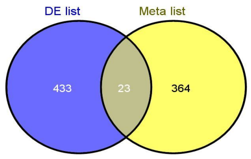	KPNB1	3837	Latency over-expressed
	RAN	5901	Latency over-expressed
	UBE2I	7329	Latency over-expressed
	PPIB	5479	Latency over-expressed
	HSPA9	3313	Latency over-expressed
	ACTB	60	Latency over-expressed
	RNPS1	10921	Latency over-expressed
	NCL	4691	Latency over-expressed
	H3F3A	3020	Latency over-expressed
	ADRBK1	156	Latency over-expressed
	KPNA2	3838	Latency over-expressed
	HNRNPA1	3178	Latency over-expressed
	FEN1	2237	Latency over-expressed
	PSMC1	5700	Latency over-expressed
	PSME3	10197	Latency over-expressed
	VCP	7415	Latency over-expressed
	PSMD6	9861	Latency over-expressed
	PRDX1	5052	Latency over-expressed
	PSMB3	5691	LTNP over-expressed
	GTF2E2	2961	LTNP over-expressed
	BRCA1	672	LTNP over-expressed
	AKT1	207	LTNP over-expressed
	CYFIP1	23191	LTNP over-expressed

DE genes overlapping with HIV interaction database[b]	Gene symbol	Gene ID	Regulation
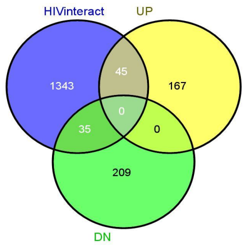	ACTB	60	Latency over-expressed
	ACTG1	71	Latency over-expressed
	APAF1	317	Latency over-expressed
	BAG1	573	Latency over-expressed
	BCL6	604	Latency over-expressed
	CALR	811	Latency over-expressed
	CAV1	857	Latency over-expressed
	CCNE1	898	Latency over-expressed
	CDC6	990	Latency over-expressed
	CDC20	991	Latency over-expressed
	CHRNA1	1134	Latency over-expressed
	CLCA1	1179	Latency over-expressed

	FEN1	2237	Latency over-expressed
	GPI	2821	Latency over-expressed
	GTF3C2	2976	Latency over-expressed
	H2AFZ	3015	Latency over-expressed
	H3F3A	3020	Latency over-expressed
	HNRNPA1	3178	Latency over-expressed
	HSPA9	3313	Latency over-expressed
	ID1	3397	Latency over-expressed
	IL1B	3553	Latency over-expressed
	IL8	3576	Latency over-expressed
	KPNB1	3837	Latency over-expressed
	KPNA2	3838	Latency over-expressed
	LRP1	4035	Latency over-expressed
	METTL1	4234	Latency over-expressed
	NCL	4691	Latency over-expressed
	NFATC4	4776	Latency over-expressed
	SERPINB2	5055	Latency over-expressed
	PIK3CA	5290	Latency over-expressed
	PLAU	5328	Latency over-expressed
	PPIB	5479	Latency over-expressed
	PRKD1	5587	Latency over-expressed
	PSMC1	5700	Latency over-expressed
	RAN	5901	Latency over-expressed
	SFRP1	6422	Latency over-expressed
	SNRPB	6628	Latency over-expressed
	SRF	6722	Latency over-expressed
	TCEB2	6923	Latency over-expressed
	UBE2I	7329	Latency over-expressed
	SUMO1	7341	Latency over-expressed
	CHST10	9486	Latency over-expressed
	PSMD6	9861	Latency over-expressed
	PSME3	10197	Latency over-expressed
	SF3B4	10262	Latency over-expressed
	ADCY7	113	LTNP over-expressed
	AKT1	207	LTNP over-expressed
	BIRC2	329	LTNP over-expressed
	BIRC3	330	LTNP over-expressed
	CCND1	595	LTNP over-expressed
	BRCA1	672	LTNP over-expressed
	CAPN3	825	LTNP over-expressed
	CASP1	834	LTNP over-expressed
	CD33	945	LTNP over-expressed
	EGR1	1958	LTNP over-expressed
	ELA2	1991	LTNP over-expressed

	ENDOG	2021	LTNP over-expressed
	GFAP	2670	LTNP over-expressed
	GTF2E2	2961	LTNP over-expressed
	HLA-DPB1	3115	LTNP over-expressed
	HLA-DQB1	3119	LTNP over-expressed
	HLA-G	3135	LTNP over-expressed
	IFNB1	3456	LTNP over-expressed
	IL6R	3570	LTNP over-expressed
	KDR	3791	LTNP over-expressed
	LAMA3	3909	LTNP over-expressed
	ABCB4	5244	LTNP over-expressed
	PRKAR2B	5577	LTNP over-expressed
	PSMB3	5691	LTNP over-expressed
	SMARCA2	6595	LTNP over-expressed
	STAT6	6778	LTNP over-expressed
	TAP1	6890	LTNP over-expressed
	TGFA	7039	LTNP over-expressed
	VCAM1	7412	LTNP over-expressed
	PCAF	8850	LTNP over-expressed
	TJP2	9414	LTNP over-expressed
	ISG15	9636	LTNP over-expressed
	NUTF2	10204	LTNP over-expressed
	APOBEC3G	60489	LTNP over-expressed
	TLN2	83660	LTNP over-expressed

[a] Reference 25-29 in text.

[b] HIV interaction database: http://www.ncbi.nlm.nih.gov/RefSeq/HIVInteractions/
