# Supplementary material for: Bridging HIV-1 Cellular Latency and Clinical Long-Term Non-Progressor: An Interactomic View
Source: PLoS One. 2013 Feb 25;8(2):e55791. doi: 10.1371/journal.pone.0055791 (PMC3581534; doi:10.1371/journal.pone.0055791)
Supplement: Table S3 — Primers used in this study. (PDF) [file pone.0055791.s004.pdf]

SUPPLEMENTARY DATA

TABLES

TableS3 Primers used in this study

Gene	Entrez Gene ID	Sequence
KPNA2	3838	5′- ACATAATCCGGGCTGGTTTGA -3’
		5′- GGGAGATGCCAACAGAGAAATG -3’
KPNB1	3837	5′- CCACTTTCCTTGTGGAACTGT -3’
		5′- CTCTGCTGATATTGTGCCTTGA -3’
ATP5G3	518	5′- ATGGTGTGTCTCAGCTAATCCA -3′
		5’- CCACTCCTACTGTTGCAGCA -3’
SRSF3	6428	5′- TGGCAACAAGACGGAATTGGA-3’
		5′- CAAAGCCGGGTGGGTTTCTA-3’
SF3B4	10262	5′- GCATCAGCTCACAACAAAAACC-3’
		5′- GCAACTTCTCATCAATCTCAGGG-3’
GAPDH	2597	5′-GTGAAGGTCGGAGTCAACG-3′
		5′-TGAGGTCAATGAAGGGGTC-3′
