# Supplementary material for: Bridging HIV-1 Cellular Latency and Clinical Long-Term Non-Progressor: An Interactomic View
Source: PLoS One. 2013 Feb 25;8(2):e55791. doi: 10.1371/journal.pone.0055791 (PMC3581534; doi:10.1371/journal.pone.0055791)
Supplement: Figure S1 — The network of latency over-expressed genes in LTNP state. (PDF) [file pone.0055791.s005.pdf]

FIGURES

Fig.S1

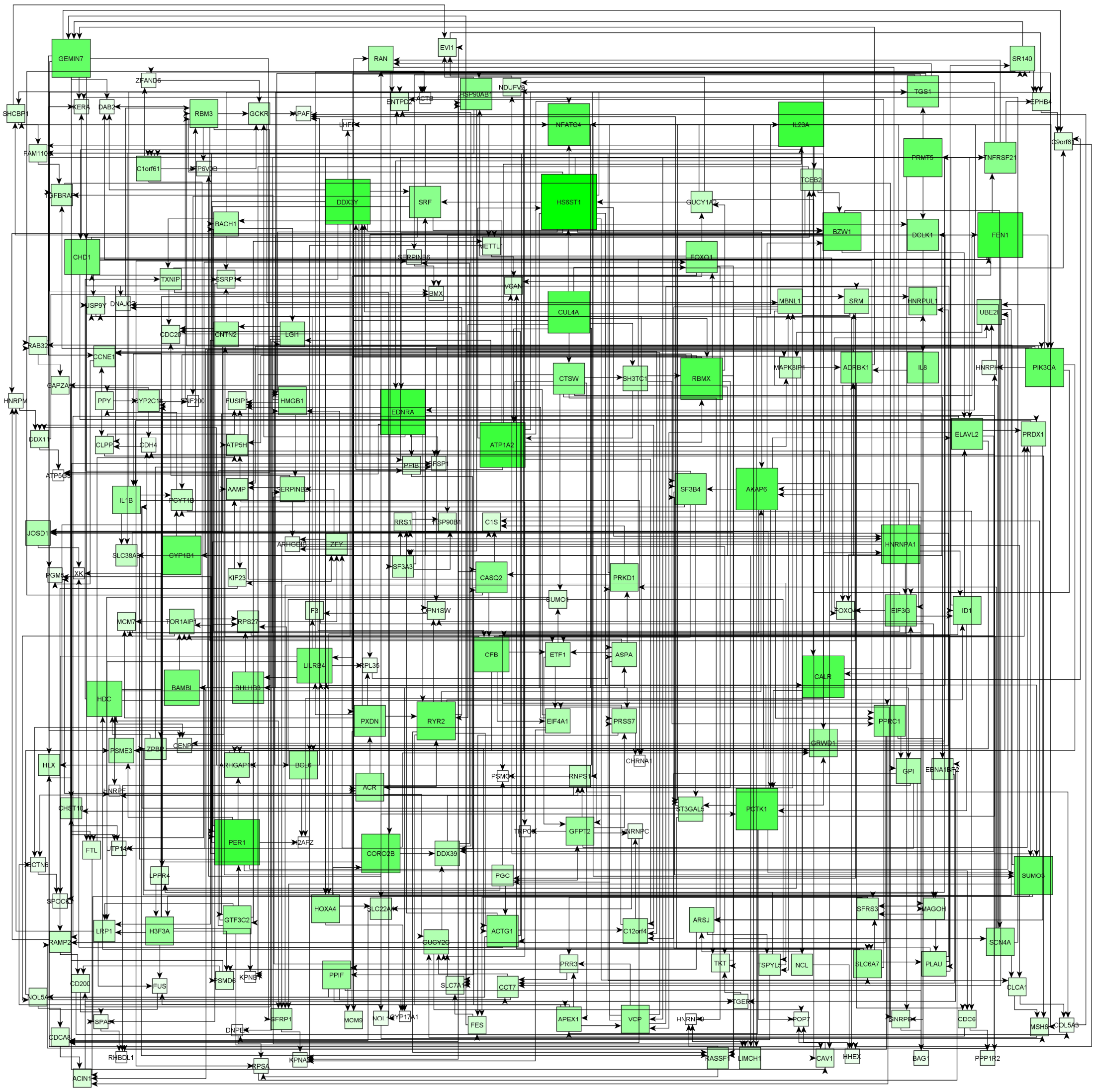

Fig.S1. Latency genes-LTNP state network. The network was inferred for 212 HIV latency over-expressed genes from all LTNP samples. Standard Banjo parameters were adopted with a q6 discretization policy. The consensus graph depicted here was obtained from the concensus of the best 100 nets (after searching through 3×10^8 networks). The node size was proportional to the betweeness centrality and visually reinforced.
